# Cytotoxic Chemotherapy and CD4+ Effector T Cells: An Emerging Alliance for Durable Antitumor Effects

**DOI:** 10.1155/2012/890178

**Published:** 2012-02-06

**Authors:** Zhi-Chun Ding, Gang Zhou

**Affiliations:** ^1^Cancer Immunotherapy Program, Cancer Center, Georgia Health Sciences University, Augusta, GA 30912, USA; ^2^Hematology/Oncology Section, Department of Medicine, School of Medicine, Georgia Health Sciences University, Augusta, GA, USA

## Abstract

Standard cytotoxic chemotherapy can initially achieve high response rates, but relapses often occur in patients and represent a severe clinical problem. As increasing numbers of chemotherapeutic agents are found to have immunostimulatory effects, there is a growing interest to combine chemotherapy and immunotherapy for synergistic antitumor effects and improved clinical benefits. Findings from recent studies suggest that highly activated, polyfunctional CD4+ effector T cells have tremendous potential in strengthening and sustaining the overall host antitumor immunity in the postchemotherapy window. This review focuses on the latest progresses regarding the impact of chemotherapy on CD4+ T-cell phenotype and function and discusses the prospect of exploiting CD4+ T cells to control tumor progression and prevent relapse after chemotherapy.

## 1. Introduction

As a major treatment modality for many advanced cancers, conventional chemotherapy can achieve high response rates but is rarely curative. The mounting evidence that many chemotherapeutic agents have immunostimulatory effects has provided a compelling rationale for developing combined chemoimmunotherapy strategy to achieve improved patient outcomes [1–3]. Current cancer immunotherapies predominantly rely on CD8+ T cells to fight against tumors. Although it is increasingly clear that proinflammatory CD4+ effector T cells are critical determinants of effective antitumor immune responses [4–9], the utilization of CD4+ T cell-based immunotherapy in combination with chemotherapy to control tumor progression and recurrence has not been fully explored. Nonetheless, a plethora of information accumulated from preclinical and clinical studies suggests that these two treatment modalities might be mutually reinforcing, and therefore their combination represents an effective chemoimmunotherapy strategy.

## 2. Anticancer Drugs and Immune Activation

Anticancer drugs are selected for their cytotoxicity toward cancerous cells. Although some anticancer drugs were known to have immune-potentiating effects long time ago [10, 11], the therapeutic potential of this property has been largely ignored. As increasing numbers of conventional chemotherapeutic agents are found to possess immunostimulatory properties, it has come to the realization in recent years that elicitation of the host antitumor immunity may constitute an integral component of the anticancer efficacy of some antineoplastic agents [12].

Multiple classes of anticancer chemotherapeutic drugs have been reported to exert immune enhancing effects, and a number of them have been extensively studied. Cyclophosphamide (CTX) is an alkylating agent chemically related to nitrogen mustard. As a prodrug, CTX is converted into its active metabolite derivative phosphoramide mustard in the liver. Phosphoramide mustard inhibits DNA replication by forming crosslinks between (interstrand) and within (intrastrand) DNA strands. CTX is often used in combination with other anticancer drugs in the treatment of lymphomas and some solid tumors. Doxorubicin is a cytotoxic anthracycline antibiotic. It is known to bind to nucleic acids by intercalating the DNA strands and disrupting DNA replication. Doxorubicin is commonly used to treat hematological malignancies (leukemia, lymphoma, and multiple myeloma), and many types of solid tumors. Gemcitabine is a pyrimidine nucleoside analog that acts as an antimetabolite. Gemcitabine is used in a wide range of carcinomas, including lung, pancreatic, breast, and bladder cancer. Paclitaxel and docetaxel belong to the taxane class of drugs that act as mitotic inhibitors. These drugs cause cell-cycle arrest by stabilizing GDP-bound tubulin in microtubules, thereby disrupting the process of cell division. They are currently used to treat patients with lung, breast, prostate, and ovarian cancer. Cisplatin and oxaliplatin are platinum-based anticancer drugs. These platinum complexes induce apoptosis in malignant cells by causing crosslinking of DNA.

Although these anticancer drugs cause tumor destruction through different mechanisms, they share some common features in exerting immune-enhancing effects.

### 2.1. Inducing Immunogenic Tumor Cell Death

Tumor cells killed by anticancer drugs not only provide the source of tumor antigens but also release “danger signals” that awaken the innate immune cells, which in turn activate the adaptive immune system. Studies from Zitvogel's group have characterized several prominent features of immunogenic cell death after cytotoxic chemotherapy, including translocation of calreticulin (CRT), secretion of high-mobility-group box 1 (HMGB1), and release of adenosine triphosphate (ATP) by dying tumor cells. These studies reported that doxorubicin induces rapid translocation of the endoplasmic reticulum-resident protein calreticulin to tumor-cell surface, presenting a “eat-me” signal for phagocytosis by dendritic cells [13]. HMGB1 released by dying tumor cells after doxorubicin or oxaliplatin treatment acts upon toll-like receptor 4 (TLR4) on dendritic cells to initiate efficient antigen processing and presentation that involves the Myd88-signaling pathway [14]. Doxorubicin and oxaliplatin can also induce release of ATP by tumor cells, which triggers purinergic P2RX7 receptors on dendritic cells (DCs) to activate the NOD-like receptor family, pyrin-domain-containing protein 3-dependent caspase-1 activation complex, namely, the NLRP3 inflammasome, which ultimately leads to IL-1*β*-dependent adaptive immunity [15]. Along the same line, cyclophosphamide has been recently reported to cause CRT translocation and HMGB1 release in some types of tumor [16, 17]. Furthermore, it has been shown that tumor-cell apoptosis induced by gemcitabine can enhance DC cross-presentation of tumor antigen to CD8+ T cells [18], but it is not yet clear whether CRT translocation, mobilization of HMGB1, and ATP are involved in the process.

### 2.2. Mitigating Immunosuppressive Mechanisms

The ability of tumors to evade immune destruction is critical for tumor formation and progression and is now regarded as an emerging hallmark of cancer [19]. Under the selection pressure imposed by natural immune surveillance or therapeutic interventions, tumors may avoid immune attacks through passive mechanisms such as downmodulating the expressions of the relevant MHC-I molecules and antigens [20, 21]. In addition, tumor cells have evolved to employ multiple immune regulatory mechanisms to actively attenuate and subvert antitumor immune responses. Regulatory T cells (Treg) and myeloid-derived suppressor cells (MDSCs) are frequently enriched in the tumor microenvironment and facilitate tumor immune evasion [22]. Some chemotherapeutic agents can potentiate antitumor immune responses by directly targeting these immunosuppressive cells. Low-dose cyclophosphamide (100 mg/kg) is capable of depleting cycling CD4+CD25+ Tregs and inhibiting their suppressive activity [23, 24]. As a result, the effector activities of cytotoxic CD8+ T cells and NK cells are unmasked to control tumor growth [25–27]. A recent study has suggested that CTX can preferentially deplete tolerogenic CD8+ lymphoid-resident DCs, leading to diminished Treg suppression and enhanced effector T-cell function as manifested by induction of concomitant immunity in a prophylactic setting [28]. It is currently unclear whether this mechanism of action is operative in a therapeutic setting. On a different note, gemcitabine does not deplete Tregs [24] but selectively reduces CD11b+Gr1+ MDSCs and enhances the antitumor activities of CD8+ T cells and NK cells [29].

### 2.3. Creating Lymphopenia and Immunogenic Milieu

Many anticancer drugs can cause varied degree of lymphodepletion [30]. It has been well established that lymphodepletion induced by chemotherapy or radiotherapy profoundly enhances the efficacy of adoptive cell therapy (ACT) and cancer vaccines [31]. This is likely due to the combined effects of creation of space and increased availability of stimulatory growth factors that lead to enhanced proliferation and survival of activated T cells [32]. In this regard, cyclophosphamide is a representative anticancer drug that causes profound lymphodepletion while creating an immune milieu rich of type I interferons (IFNs) and common gamma-chain cytokines (IL2, IL7, and IL15) [33, 34]. Of notice, type I interferons are known to promote DC maturation and T-cell differentiation [35–38]. IL7 is essential for survival and memory formation of tumor-reactive T cells, and neutralization of IL7 after CTX administration diminishes the number of tumor-reactive T cells in an adoptive transfer model [33]. Besides strengthening the activities of immune cells, chemotherapy also promotes the trafficking of activated immune cells to the sites of tumor [33, 39, 40]. Accumulating evidence demonstrates that there is a surge of proinflammatory cytokines/chemokines, such as GMCSF, IL1*β*, IL6, and CXCL10, in the postchemotherapy immune milieu, which may contribute to the recruitment and retention of tumor-reactive immune cells, including activated CD8+ and CD4+ T cells, DCs, macrophages, and neutrophiles, in the tumor microenvironment [15, 17, 34].

### 2.4. Sensitizing Tumor Cells to Immune Destruction

In addition to attracting activated immune cells to the tumor loci, chemotherapy may render tumor cells more susceptible to immune attack. It has been shown that doxorubicin, cisplatin, and paclitaxel can sensitize tumor cells to the cytolytic effect of CD8+ T cells by making them permeable to granzyme B via mannose-6-phosphate receptors on the surface of tumor cells [40]. Moreover, chemotherapy with cyclophosphamide can sensitize tumor cells to TRAIL-dependent CD8+ T cell-mediated immune destruction [41].

## 3. Chemotherapy and Antitumor CD4 Responses

A great deal of effort has been focused on understanding how chemotherapy potentiates CD8+ T-cell responses [27, 38, 41, 42], mitigates Treg-mediated immune suppression [23, 26, 43], and enhances antigen presentation [13, 14, 28, 44, 45]. Although tumor-reactive CD4+ effector/helper T cells are increasingly recognized as critical determinants of effective antitumor immune responses, the effect of chemotherapy on these cells is largely neglected, and the role of CD4+ T cells in modulating postchemotherapy host immunity is almost entirely unknown. In the following we mainly focus on findings that concern the impact of chemotherapy on the interactions between tumors and CD4+ T cells.

### 3.1. CD4+ T Cells Subsets and Their Diverse Roles in Tumor Immunity

Upon stimulation naïve CD4+ T cells differentiate into effector cells known as T helper (Th) cells. Originally Th cells were classified into Th1 and Th2 lineages, depending on the cytokine profiles of the effector cells [46]. With the discovery of new T-cell lineages in recent years, the Th1/Th2 paradigm has been revised to reflect a much broadened spectrum of CD4+ T-cell subsets. It has now been established that naïve CD4+ T cells can differentiate into four major lineages, including Th1, Th2, Th17, and Treg cells [47], and that Th cells are plastic—cells of one lineage can be converted to another lineage under certain circumstances [48].

The distinct CD4+ T-cell subsets have varied impact on tumor growth. Th1 cells, characterized by production of IFN*γ* and TNF*α*, often lead to enhanced activation of cytotoxic CD8+ T cells, DCs and macrophages, exhibiting beneficial antitumor effects. In contrast, IL4-producing Th2 cells may promote tumor progression by enhancing the activity of protumor macrophages [49] although Th2 cells can also mediate tumor rejection under certain condition [50]. Currently there is much debate about the role of Th17 cells in antitumor immunity [51], because both tumor rejection and tumor promotion involving Th17 cells and their major product proinflammatory cytokine IL17 have been reported [52–55]. Treg cells act to dampen antitumor immunity by suppressing the effector functions of a variety of immune cells, including Th1 cells [56–58], CD8+ T cells [5], NK cells [59], and tumor-infiltrating DCs [60].

### 3.2. Effect of Chemotherapy on CD4+ T-Cell Effector Development

So far, among the aforementioned anticancer drugs, cyclophosphamide (CTX) appears to be the most effective one in enhancing antitumor CD4 responses, particularly when used in combination with adoptive cell therapy (ACT). It has been demonstrated in various preclinical models that CTX treatment followed by adoptive transfer of tumor-reactive CD4+ T cells, either monoclonal T-cell clones derived from TCR-transgenic mice, or activated polyclonal CD4+ T cells derived from preimmunized mice, leads to eradication of established tumors [61–64]. One salient observation from these studies is that the robust antitumor effects are associated with the development of Th1 antitumor immunity. In line with an early study showing that CTX induced a Th2 to Th1 shift in the cytokine profile of lymphoma-bearing rats [65], we have recently reported in a mouse lymphoma model that CTX overcomes tumor-driven aberrant CD4+ T-cell differentiation and directs CD4+ T cells to become highly activated polyfunctional effector cells, marked by their ability to concomitantly produce multiple Th1-type cytokines including IL2, IFN*γ*, and TNF*α* [64]. In a mouse melanoma model, Quezada et al. reported that tumor-specific CD4+ T cells acquired a similar polyfunctional phenotype in postradiotherapy hosts [66], suggesting that the immunogenic milieu created by chemotherapy or radiotherapy may share some common features in terms of driving CD4+ T-cell effector differentiation. In addition to promoting Th1 differentiation, there is emerging evidence that CTX also induces Th17 cells [34, 67]. These Th17 cells are likely de novo induced in the postchemotherapy milieu, because they are not converted from Treg cells [67], and do not coexpress IFN*γ* [34]. In contrast, doxorubicin and oxaliplatin each induces IL17-producing *γδ*T cells but not Th17 cells [68]. It will be of interest to test additional anticancer drugs to define the common features of the drugs that are capable of driving effector CD4 responses like CTX.

### 3.3. Mechanisms by Which Anticancer Drugs Modulate CD4 Responses

Even though CTX is by far the most potent CD4-potentiating anticancer drug demonstrated experimentally, the cellular and molecular mechanisms underlying its effect are not well understood. In addition to its well-known effect of depleting suppressor T cells, accumulating evidence has established a link between productive CD4+ T-cell responses and an immunogenic milieu induced by CTX [17, 33, 36, 64]. The immunogenic milieu is rich of various growth factors and proinflammatory cytokines and chemokines, among which type I IFNs and IL7 have been shown to exert particularly important immunostimulatory effects. Type I IFNs can augment immune responses through enhanced stimulation of dendritic cells [69]. It has been shown that DCs require type I IFNs to mature and induce CD4+ Th1 immunity [70]. In the same vein, a recent study has reported that IFN*α* enhances T helper cell functions while reducing Treg activity through modulating APC activation [71]. In addition to supporting T-cell survival and homeostasis, IL7 has recently been shown to antagonize cbl-b and TGF*β* signaling, two pathways involved in inhibiting T-cell activation, leading to augmented Th17 differentiation [72]. Moreover, it has been reported that IL7 promotes Th1-like immunity and inhibits Treg activity [73, 74]. Altogether it is conceivable that CTX's multifaceted and dynamic immunomodulatory effects, for example, depletion of Treg, creation of lymphopenia, and induction of stimulatory cytokines superimpose to foster a profoundly immunogenic milieu that drives the development of fully differentiated Th1 or Th17 effector T cells. To better understand the mechanisms underlying the diverse CD4+ T-cell differentiation in postchemotherapy setting, future studies should dissect the interrelation of the above-mentioned contributing factors, and their relative contribution to the functional development of tumor-specific CD4+ T cells.

### 3.4. Antitumor Effects of CD4+ Effector T Cells

#### 3.4.1. Activating Tumoricidal CD8 and Macrophages and Sensitizing Tumor Stroma

CD4+ T cells have been regarded as specialized helper cells that assist in the activation of other innate and adaptive immune cells. Once properly activated, CD4+ T cells express an array of effector molecules, including CD40L, IL2, IFN*γ*, and TNF*α*, which play critical roles in orchestrating effective antitumor immune responses. Consistent with the well-defined role of CD40L in transmitting CD4 help for CD8+ T cells [75–77], it has been shown in different animal models that activated CD4+ T cells can license DCs in the tumor microenvironment via CD40L-CD40 interaction, leading to priming of tumor-reactive CD8+ T cells which in turn mediate long-term protection [78, 79]. In addition to licensing of DC, some previously unappreciated help activities of CD4+ T cells have recently been uncovered, revealing the molecular basis of the once vaguely-defined “post-licensing” role of CD4+ T cells [80]. For example, it has been shown that CD4+ effector T cells recruit activated CD8+ T cells via the action of IFN*γ* [81, 82] and promote CD8+ T-cell cytolytic function and proliferation through IL2 [82]. Besides targeting tumor cells, CD4+ effector T cells have been implicated in inhibiting tumor angiogenesis by acting on tumor stroma via IFN*γ* [83]. Given that CD8+ T cell-derived TNF*α* and IFN*γ* can sensitize tumor stroma and mediate bystander tumor eradication [84], we speculate that polyfunctional CD4+ effector T cells have the same effect because these cells can produce these two cytokines simultaneously [34]. Notably, it has been reported that Th1-derived IFN*γ* also renders macrophages cytotoxic to cancer cells [6, 85]. Interestingly, Beatty et al. reported that CD40-activated macrophages become tumoricidal and facilitate the destruction of tumor stroma in mice and humans with pancreatic carcinoma [86]. Although this study used an agonist CD40 antibody to activate macrophages, it is tempting to speculate that CD40L-expressing CD4+ effector T cells would achieve similar effects.

#### 3.4.2. Conditioning a Protective Inflammatory Milieu

Chemotherapy often induces inflammation in the tumor microenvironment by causing tumor cell death and tissue damage. Paradoxically, many of the proinflammatory cytokines induced after chemotherapy, particularly IL1*β*, IL6, and IFN*α*/*β*, can exert both tumor-inhibiting and tumor-promoting effects (double-edged sword) [87, 88]. On one hand, IFN*α*/*β* and IL1*β* both can directly act on CD4+ T cells to enhance their activation and differentiation [89–92]. In addition, IFN*α*/*β* and IL1*β* can augment antigen presentation and facilitate priming of T cells [15, 37, 70, 93]. Moreover, IL6 and IFN*α*/*β* can potentiate effector cells to resist Treg-mediated suppression [71, 94], and IL6 and IL1*β* can mediate Treg→Th17 conversion [95–97]. On the other hand, IL1*β* and IL6 have been shown to drive tumorigenesis [98–103] and dampen host immunity by expanding myeloid-derived suppressor cells (MDSCs) [104–106]. IFN*α*/*β* are potent inducers of coinhibitory molecules PDL1 [107] and PD1 [108], and immunosuppressive enzyme indoleamine 2,3-dioxygenase (IDO) [109, 110]. Furthermore, it has been shown that IL6 contributes to chemoresistance [111]. Intriguingly, the efficacy of many cancer therapies is often associated with certain degree of inflammatory responses [34, 112, 113]. A recent study by Haabeth et al. has suggested that unopposed inflammation may promote tumor progression while the presence of Th1 cells can tilt inflammation toward effective antitumor immunity [85]. This hypothesis is supported by the observation that chronic inflammation associated with psoriasis, a Th1-mediated autoimmune disease affecting the skin, does not promote the development of skin cancers [114]. Therefore, it is likely that Th1 CD4+ T cells play a critical role in conditioning a tumor-inhibiting inflammatory milieu that facilitates immune activation and tumor destruction.

#### 3.4.3. Mediating Direct Tumor Destruction

Besides rendering other immune cells tumoricidal, CD4+ T cells have the capability to mediate direct tumor destruction. It has been shown that CD4+ T cells can induce apoptosis in tumor cells through FAS- or TRAIL-dependent pathway [115, 116]. Moreover, there is accumulating evidence that CD4+ T cells can acquire cytolytic activities like cytotoxic CD8+ T cells [117–121]. However, the significance of this property has been largely ignored, until recently two studies have provided compelling evidence that cytotoxic CD4+ T cells developed in a lymphopenic environment can eradicate established melanoma as a result of direct killing of the tumor cells through granzyme B [66, 122]. Currently it is unknown whether cytotoxic CD4+ T cells and helper CD4+ T cells develop in parallel, or they are the same cells at different stages of differentiation. Nevertheless, Qui et al. provided evidence that costimulation through CD134 (OX40) and CD137 (4-1BB) is required to drive the differentiation of cytotoxic CD4+ effector cells in an eomesodermin-dependent manner [123]. Although cytotoxic CD4+ and CD8+ T cells appear to mediate tumor killing using the same effector molecules, such as granzyme B and perforin, they target MHC-II and MHC-I-restricted antigens, respectively. One important implication of CD4+ T-cell cytotoxicity is that CD4+ T-cell-mediated tumor destruction may result in antigen spreading, which is associated with broadened antitumor CD8 responses and improved clinical responses [8, 124–126].

In summary, with an arsenal of diverse cancer-fighting weapons, CD4+ T cells can mediate tumor destruction either on their own or by cooperating with other immune cells. Whereas CD4+ T cells alone clearly have the potential to effectively eradicate tumors [66, 122, 127], the majority of published studies indicate that the optimal antitumor effects are achieved when CD4+ T cells act in concert with tumor-reactive CD8+ T cells [8, 78–80, 128–133], macrophages [6], or NK cells [7]. A long-held perception is that CD4 antitumor immunity is only relevant to the treatment of MHC-II^+^ tumors. Nevertheless, due to the wide-range mode of actions, CD4+ T cells have been shown to play active and indispensable roles in controlling both MHC-II^+^ [63, 64] and MHC-II^−^ tumors [6, 7, 78, 79, 127, 134, 135]. It is worth noting that some solid tumors, melanoma, for instance, can be induced to express MHC-II upon encountering IFN*γ* and thus become direct targets of CD4+ effector T cells [66, 122]. Therefore, the generation of effective CD4+ T-cell responses has great therapeutic potential and broad clinical relevance.

## 4. Inhibitory Mechanisms That Attenuate Antitumor CD4+ T-Cell Responses

Tumor-specific CD4+ T cells are subject to a variety of tolerizing mechanisms operative in the tumor microenvironment. Induction of anergy in antigen-specific CD4+ T cells is an early event in the course of tumor progression [136]. We and others show that tumor-antigen recognition is accompanied with induction of both CD4+ effector cells and Tregs [56, 57, 137]. However, the tolerogenic nature of the tumor milieu progressively renders CD4+ effector T cells dysfunctional, characterized by sustained expression of PD1 and heightened apoptosis [64]. Thus, the anergic phenotype of the overall CD4 population represents the net result of Treg induction, effector dysfunction, and active immune suppression. Treg cells enriched in tumor may come from expansion of preexisting Tregs, and de novo induction of Treg cells [137, 138], which may occur in both antigen-dependent [138] and -independent [139] manner in tumor-bearing hosts. Pertaining to combinatory chemoimmunotherapy, it will be important to determine if highly activated CD4+ effector T cells are susceptible to Treg conversion in the face of minimal residual disease after chemotherapy. Although it has been shown that polarized Th1 effector cells and memory CD4+ T cells are refractory to conversion to Tregs [140, 141], whether this is the case in the postchemotherapy setting is yet to be addressed.

Tregs have been shown to attenuate antitumor responses through a variety of mechanisms, including deactivating DCs [142, 143], preventing CD8+ T-cell-mediated cytolysis [144], and direct killing of DC, NK, and CD8+ T cells [145, 146]. Importantly, Tregs may operate in concert with other regulatory mechanisms, including MDSC, coinhibitory molecule PD1, and immunosuppressive enzyme IDO, to form a self-reinforcing immunosuppressive network, posing a severe threat to the magnitude and durability of an effective antitumor immune response. MDSCs can act as tolerogenic APCs to expand Tregs [147, 148]. IDO+ DCs can directly activate Tregs which subsequently mediate suppression in a PD1/PDL1-dependent fashion [149]. Programmed death 1 (PD1) was initially found to mediate CD8+ T-cell functional exhaustion during chronic viral infections [150]. Subsequent studies confirmed the existence of exhausted PD1^high^ CD8+ T cells during tumor progression [151, 152]. However, the role of PD1 in regulating CD4+ T-cell response in the tumor context is less clear. Using a mouse B-cell lymphoma model, we provided clear evidence that PD1^high^ CD4+ T cells constituted a fraction of tumor antigen-experienced cells and were associated with downregulation of IL7 receptor and elevated level of apoptosis [64]. Interestingly, we showed in this model that PD1 was not required for tumor-driven Treg induction, while two other studies reported that PDL1 was involved in peripheral Treg induction and maintenance [153, 154]. Given that PD1 is not the only receptor for PDL1 [155–157], the seemingly discrepant results suggest that PDL1 on DCs may differentially regulate Treg induction and effector T-cell dysfunction through engaging different receptors on CD4+ T cells. This is supported by the observation that PD1 and Foxp3 have a nonoverlapping expression pattern in CD4+ T cells infiltrating B-cell lymphoma [158, 159]. Collectively, these findings and the results from other studies [160–163] support a scenario in which Treg-mediated suppression and PD1-dependent T-cell dysfunction contribute independently but synergistically to the failed immunological control of tumor growth.

## 5. Implications for Combined Chemoimmunotherapy

Standard chemotherapy is a major treatment option for many types of cancer. It can effectively treat the symptom of cancer initially, but frequently its efficacy is compromised by late tumor recurrence. The ability of some anticancer drugs to drive productive CD4+ T-cell responses, and the versatile and pivotal roles of CD4+ effector T cells in mediating antitumor effects, provide strong rationales for developing a strategy that utilizes CD4+ effector T cells to strengthen and sustain the postchemotherapy antitumor immunity. This can be achieved clinically through the combination of chemotherapy and adoptive immunotherapy or therapeutic vaccination. Indeed, the efficacy of this strategy has been hinted by some elegant clinical studies, which showed that better immunological and clinical responses were obtained in melanoma or myeloma patients that had received CD4+ T cell-containing donor cells following preconditioning chemotherapy [129, 131].

To overcome tumor-induced immune tolerance, additional maintenance regimens are needed to keep CD4+ T cells in the polyfunctional effector state. Many of the currently available immune modulators [164], such as recombinant IL7, CD40 agonist, PD1 blockade, and CTLA4 blockade, can be applied to potentiate and sustain CD4 effectors in addition to enhancing antitumor CD8 responses. We showed that polyfunctional CD4+ T cells have the unusual distinguishing attribute of high levels of IL7 receptor expression [64], suggesting that these cells can be preferentially expanded by supplying exogenous IL7. Moreover, activating DCs with an anti-CD40 agonist antibody can prevent CD4+ T-cell tolerance [165]. PD1 blockade, currently undergoing extensive clinical trials for a variety of cancers [166, 167], is largely expected to restore CD8+ T-cell antitumor function but may as well benefit CD4+ effector T cells. Notably, CTLA4 blockade with ipilimumab, recently approved by FDA for the treatment of late-stage melanoma, has been shown to promote the generation of polyfunctional CD4+ T cells in response to vaccination [168].

With regard to alleviating Treg-mediated immunosuppression, current approaches only have limited success in therapeutic settings. Low-dose CTX reduces and inactivates Tregs, but doing so only transiently. Application of denileukin diftitox (Ontak) did not result in consistent clinical outcomes [169, 170], likely due to its effect on both effector T cells and Tregs. Findings from some recent studies suggest new strategies for disarming Tregs. It has been shown that combined use of CTX and an agonist antibody targeting the costimulatory receptor OX40 can result in intratumoral apoptosis of Tregs [42]. Moreover, Sharma et al. reported that disrupting the IDO pathway with clinically applicable pharmacological inhibitors can reprogram Tregs to Th17 cells [171].

Altogether, a successful combined chemoimmunotherapy should integrate strategies that target multiple mutually reinforcing immune pathways that converge to attain productive CD4 effector responses, thereby maintaining a durable and effective antitumor immunity after chemotherapy.

## 6. Conclusions

**Figure 1:**
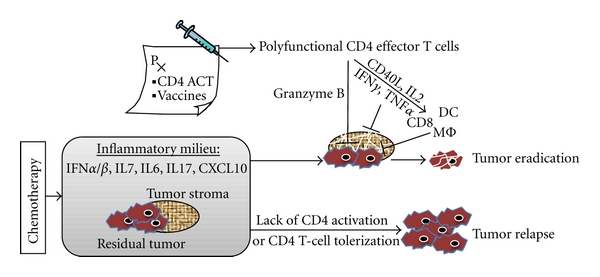
Hypothetical model of the mutually reinforcing effect of chemotherapy and antitumor CD4+ effector T cells. Chemotherapy reduces tumor burden, releases tumor antigens, and induces inflammation. In this highly immunogenic milieu created after chemotherapy, therapeutic immunological maneuvers such as adoptive cell therapy (ACT) using tumor-reactive CD4+ T cells or cancer vaccines can lead to the generation of highly activated CD4+ effector T cells with polyfunctional activities. These CD4+ effector T cells act as the “gatekeepers” of the overall antitumor immunity in postchemotherapy hosts, by helping the activation and function of other immune cells (CD8, DC, and macrophage) and directly attacking the tumor cells. In addition, cytokines produced by CD4+ effector T cells (IFN*γ* and TNF*α*) may also target and destroy tumor stroma and thus inhibit tumor angiogenesis. These diverse immune responses superimpose to effectively eradicate residual tumors. In contrast, without properly activated CD4+ effector T cells, an effective host antitumor immunity may not be elicited or is not sustainable, leading to tumor persistence and eventual relapse.

Although the concept of combined chemoimmunotherapy for cancer can be dated back to at least three decades ago [10, 11], its clinical application started to gain momentum only in recent years when the mechanistic basis for the synergy between chemotherapy and immunotherapy began to be unveiled at the cellular and molecular level. The emerging evidence that chemotherapy can profoundly drive the effector development of tumor-specific CD4+ T cells implicates a new direction for chemoimmunotherapy, which aims to capitalize on the antitumor potential of CD4+ effector T cells. In light of the unique and pivotal roles of tumor-reactive CD4+ effector T cells, we propose a scenario in which CD4+ effector T cells act as the “gatekeepers” of the overall host antitumor immunity after chemotherapy, whose functional status (polyfunctional versus tolerized) critically determines the outcome between eradication versus recurrence of residual tumors (Figure 1). Further studies are needed to explore additional CD4+ T cell-potentiating anticancer drugs and establish clinically applicable strategies for maximum utilization of the synergy between chemotherapy and antitumor CD4 effector responses in order to achieve durable therapeutic efficacy.
